# An Interesting Imaging Presentation of a Common Benign Entity: Fibrocystic Changes in a Postmenopausal Patient

**DOI:** 10.7759/cureus.36292

**Published:** 2023-03-17

**Authors:** Brittany Q Dang, Brittany Miles, Peter Young, Jing He, Quan D Nguyen

**Affiliations:** 1 Radiology, University of Texas Medical Branch, Galveston, USA; 2 Pathology, University of Texas Medical Branch, Galveston, USA; 3 Radiology, Baylor College of Medicine, Houston, USA

**Keywords:** polycystic ovarian syndrome, postmenopause, benign breast condition, breast pathology, fibrocystic breast changes

## Abstract

Fibrocystic changes (FCCs) are common, often benign, breast lesions characterized by adenosis, fibrosis, and cyst formation. These changes are believed to be associated with fluctuating hormone levels and are predominantly found in premenopausal women due to higher levels of estrogen. Certain conditions that cause hormonal imbalances, such as polycystic ovarian syndrome, have also been associated with an increased risk of FCCs. FCCs can occur in postmenopausal women on hormonal replacement therapy but are otherwise extremely rare. Although this condition is primarily considered benign, complex cysts presenting in a rare demographic warrant further evaluation beyond screening mammograms to exclude the possibility of malignancy. In this paper, we present the case of new FCCs in a postmenopausal woman and explore the radiology, histology, carcinogenic potential, treatment options, and potential contributing factors of the condition.

## Introduction

Fibrocystic changes (FCCs) are considered the most common type of benign breast disease, with an estimated lifetime prevalence in women of up to 70% to 90% [1,2]. They encompass several clinical manifestations, including breast pain, solid masses, multiple cysts, and diffuse nodularity. FCCs typically arise in the terminal ductal lobular units in the upper outer quadrants. They are histologically characterized by fibrosis with hyalinized stroma and cysts of varying size with microcalcifications, adenosis, and epithelial hyperplasia. They are considered to be common, benign changes that do not indicate a pathologic disease but instead represent breast tissue response to the fluctuating levels of hormones throughout a woman’s life. The fluctuating levels of hormones during menstruation that cause an imbalance in estrogen and progesterone contribute to the frequency with which FCCs are seen in premenopausal women. However, the exact cause of these changes remains unclear. Certain conditions that cause hormonal imbalances, such as polycystic ovarian syndrome (PCOS), have also been associated with an increased risk of FCCs [3,4]. Evaluation of FCCs is typically performed by mammography, ultrasonography, and biopsy.

Mammography of FCCs can mimic other pathologic conditions as they may present with nonspecific findings such as nodular thickening, microcalcifications, or cysts. FCCs possess features similar to fibroadenomas but often lack the same well-circumscribed borders. Since the sensitivity of mammography may be reduced due to breast tissue density, ultrasound is the most accurate method for detecting cystic lesions. Ultrasound will typically show prominent fibroglandular tissue without a defined mass as well as well-defined cystic structures [5]. MRI is another imaging modality used to detect FCCs; however, apocrine metaplasia may present with non-mass enhancement and kinetic features suspicious for malignancy - often leading to unnecessary biopsy [6].

The physical exam may reveal only a palpable mass or breast tenderness. Most cases are found in premenopausal women between the ages of 20 to 50 years and can sometimes occur in postmenopausal women on hormone replacement therapy [7,8]. The Women’s Health Initiative demonstrated that postmenopausal women on hormonal therapy consisting of estrogen and progestin had a 74% increase in FCCs over five years, and anti-estrogen therapy resulted in a 28% decrease in FCCs in both premenopausal and postmenopausal women [9,10]. The development of new FCCs in postmenopausal women not receiving estrogen replacement therapy is extremely rare. Herein, we present a rare case of a postmenopausal female with a complex benign mass due to FCCs.

## Case presentation

**Figure 1:**
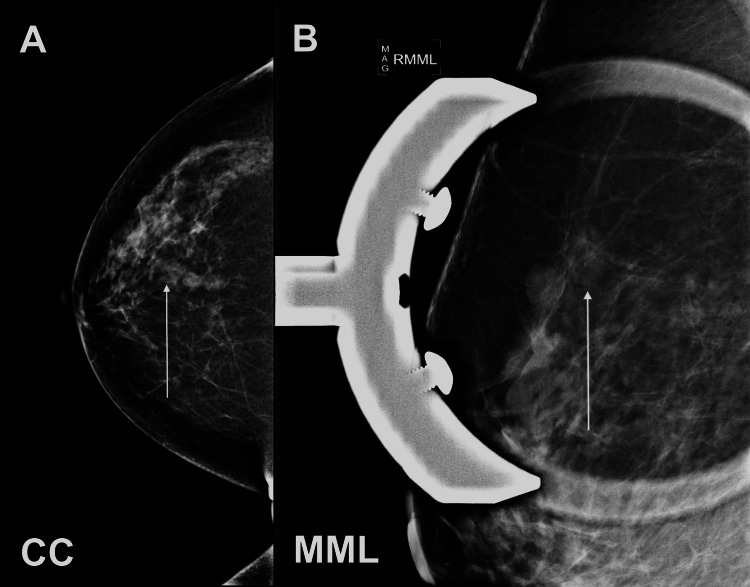
Mammogram of the right breast demonstrates an irregular mass with equal density and circumscribed borders in the upper central region, middle depth, 5 cm from the nipple (A) The craniocaudal (CC) and (B) magnified mediolateral (MML) views are presented.

**Figure 2:**
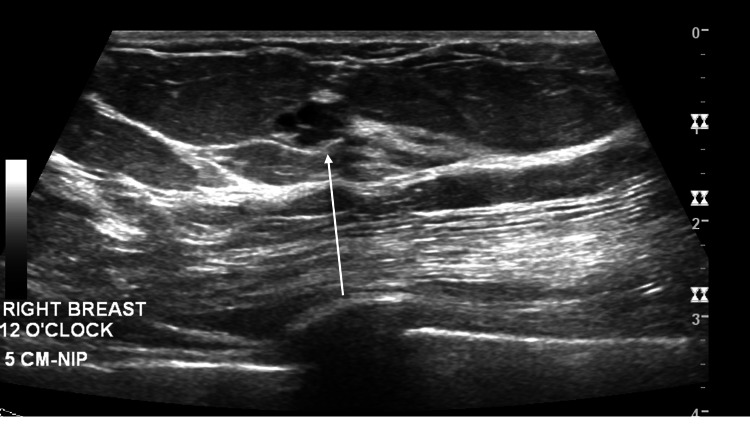
Ultrasonography of the right breast demonstrates a 7 x 7 x 5 mm solid and cystic hypoechoic mass with indistinct margins with no significant posterior features at 12 o’clock, 5 cm from the nipple

A 53-year-old postmenopausal woman with a past medical history of PCOS was found to have an abnormality in her right breast on a screening mammogram, which was new from her last mammogram five years prior. She was asymptomatic, and the physical exam was negative for any findings. She also denied any recent trauma, prior breast surgery, a family history of breast cancer, or hormone replacement therapy. The patient was called back for diagnostic mammography and ultrasound for further evaluation. Mammogram demonstrated an equal density oval mass with complex solid and cystic components on ultrasound in the right breast at 12 o’clock, 5 cm from the nipple (Figures 1, 2). The suspicious lesion was classified as Breast Imaging Reporting and Data System (BI-RADS) category 4B (>10% to <50% likelihood of malignancy), and an ultrasound-guided biopsy was recommended.

 

**Figure 3:**
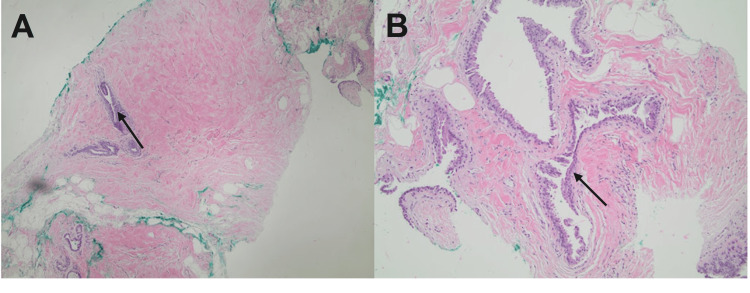
Histological finding of ultrasound-guided core biopsy of the right breast (A) The low power magnification shows dense stromal fibrosis (magnification: 40x). (B) The ductal epithelium demonstrates features of apocrine metaplasia with abundant granular eosinophilic cytoplasm and apical snouts (magnification: 100x). The breast ductal epithelium with apocrine metaplasia in the background of dense stromal fibrosis represents the feature of fibrocystic changes in the breast.

An ultrasound-guided core needle biopsy was performed, and the diagnosis of benign FCCs with stromal fibrosis, apocrine metaplasia, and columnar cell change without atypia was made (Figure 3). No malignancy was identified. Because the lesion was not detectable on physical exam, a fiduciary was placed near the site of the biopsy for future localization. No treatment was required, and the patient’s follow-up imaging was negative for malignancy over the next six years.

## Discussion

FCCs are a common, often non-cancerous, breast condition in premenopausal women or women receiving hormone replacement therapy. This case is unique as the patient is a postmenopausal woman who was not receiving hormone replacement therapy.

FCCs are rarely seen in postmenopausal women who are not on hormone replacement therapy because these changes often occur in states of increased estrogen and decreased progesterone levels, and postmenopausal women typically maintain low levels of estrogen [11,12]. Imbalances due to hyperestrogenism relative to progesterone are believed to cause dilation of ducts and vessels, stromal tissue overgrowth, and fluid retention over time, resulting in the swelling and edema often seen in FCCs. It is important to note that women who develop FCCs before menopause will often experience a decrease in these changes or complete resolution after menopause due to decreased estrogen levels [12].

In conditions such as PCOS, hyperandrogenism can cause inhibitory effects on progesterone and a consequential increase in mammary epithelial tissue, breast growth, and fibrocystic breast formation [13,14]. One study found that PCOS increased the incidence of FCCs by at least three-fold in women aged 17-36 [3]. The hormonal imbalance of PCOS has been shown to persist past menopause and contribute to lifelong inflammatory, metabolic, and cardiovascular risks [15,16]. It is possible that the lasting hormonal imbalance from PCOS may also contribute to the development of rare new postmenopausal FCCs.

Regarding malignant potential, most FCCs are considered benign; however, the histologic characteristics of the lesion appear to be an important factor in determining the risk of malignancy. FCCs may be histologically broken down into non-proliferative, proliferative without atypia, and proliferative with atypia. Proliferative FCCs are associated with usual ductal hyperplasia, while non-proliferative FCCs are not. A very small percentage of FCCs are found to be proliferative with atypia, and this feature has been linked to the development of breast cancer with a relative risk of 4.24 [17]. Moreover, FCCs with accompanying atypical ductal hyperplasia may have an increased risk for the development of ductal carcinoma in situ and mammary carcinoma. Incidental findings of FCCs on breast cancer biopsies are also possible if the FCCs develop in close proximity to a site of malignant transformation.

Even when benign FCCs are suspected based on radiographic appearance, a biopsy may be recommended to rule out malignancy in complicated cysts [18]. Additionally, when multiple cysts or clustered microcysts are present, particularly in postmenopausal women, a more thorough workup and potential biopsy are appropriate as ductal carcinoma in situ may appear radiographically similar [19]. If benign FCCs are confirmed via biopsy, and the patient is asymptomatic, no further treatment is required; postmenopausal women with FCCs may require close surveillance at six to twelve-month intervals. Symptomatic patients presenting with breast pain may be treated with non-steroidal anti-inflammatory drugs. Tamoxifen, bromocriptine, or danazol may be prescribed for patients who are symptomatic for over six months. If medications are not sufficient for symptomatic relief, needle aspiration may be performed [20].

## Conclusions

Although FCCs are most common in premenopausal women or postmenopausal women on hormone replacement therapy, they may also rarely present in postmenopausal women, not on hormone replacement therapy. However, due to the overlapping radiographic findings of FCCs and malignancy, any new finding in the postmenopausal patient should raise the consideration of biopsy for definitive diagnosis. The new development of FCCs in this patient can help add to the body of knowledge of FCCs in postmenopausal women, not on hormone replacement therapy.
